# Monogenic diabetes clinic (MDC): 3-year experience

**DOI:** 10.1007/s00592-022-01972-2

**Published:** 2022-09-30

**Authors:** Novella Rapini, Patrizia I. Patera, Riccardo Schiaffini, Paolo Ciampalini, Valentina Pampanini, Matteoli M. Cristina, Annalisa Deodati, Giorgia Bracaglia, Ottavia Porzio, Rosario Ruta, Antonio Novelli, Mafalda Mucciolo, Stefano Cianfarani, Fabrizio Barbetti

**Affiliations:** 1 Diabetology and Growth Disorders Unit, Bambino Gesù Children’s Hospital, IRCCS, 00164 Rome, Italy; 2 Clinical Laboratory Unit, Bambino Gesù Children’s Hospital, Piazza S Onofrio 4, 00165 Rome, Italy; 3 Department of Experimental Medicine, Univerisity of Rome ‘Tor Vergata’, 00131 Rome, Italy; 4 Translational Cytogenomics Research Unit, Bambino Gesù Children’s Hospital, IRCCS, 00146 Rome, Italy; 5 Department of Systems Medicine, University of Rome ‘Tor Vergata’, 00131 Rome, Italy; 6 Department of Women’s and Children’s Health, Karolinska Institutet, 17177 Stockholm, Sweden

**Keywords:** Monogenic diabetes, GCK, HNF1A, INSR, Glibenclamide, SGLT2i

## Abstract

**Aim:**

In the pediatric diabetes clinic, patients with type 1 diabetes mellitus (T1D) account for more than 90% of cases, while monogenic forms represent about 6%. Many monogenic diabetes subtypes may respond to therapies other than insulin and have chronic diabetes complication prognosis that is different from T1D. With the aim of providing a better diagnostic pipeline and a tailored care for patients with monogenic diabetes, we set up a monogenic diabetes clinic (MDC).

**Methods:**

In the first 3 years of activity 97 patients with non-autoimmune forms of hyperglycemia were referred to MDC. Genetic testing was requested for 80 patients and 68 genetic reports were available for review.

**Results:**

In 58 subjects hyperglycemia was discovered beyond 1 year of age (Group 1) and in 10 before 1 year of age (Group 2). Genetic variants considered causative of hyperglycemia were identified in 25 and 6 patients of Group 1 and 2, respectively, with a pick up rate of 43.1% (25/58) for Group 1 and 60% (6/10) for Group 2 (global pick-up rate: 45.5%; 31/68). When we considered probands of Group 1 with a parental history of hyperglycemia, 58.3% (21/36) had a positive genetic test for *GCK* or *HNF1A* genes, while pick-up rate was 18.1% (4/22) in patients with mute family history for diabetes. Specific treatments for each condition were administered in most cases.

**Conclusion:**

We conclude that MDC **may** **contribute** to provide a better diabetes care in the pediatric setting.

**Supplementary Information:**

The online version contains supplementary material available at 10.1007/s00592-022-01972-2.

## Introduction

Polygenic, autoimmune type 1 diabetes mellitus (T1DM) is the main cause of pediatric diabetes, but a pathogenic variant in a single gene can be identified in a sizeable number of patients referred to the pediatric diabetes clinic. The latter group of patients is affected by Mendelian forms of diabetes (i.e. autosomal dominant, autosomal recessive and X-linked) defects collectively known as "monogenic diabetes" mellitus (MDM) [1–5].

Genes involved in MDM have now surpassed the number of **50** and new genes are discovered at an amazing pace. The list includes "common", non-syndromic forms as well as rare, syndromic subtypes. There are two other genetic forms of diabetes not strictly under the MDM definition but considered part of this group of diseases: chromosome 6 aberrations and mutations of mitochondrial DNA. Chromosome 6 defects include uniparental paternal unidisomy, microduplications and methylation defects (collectively known **as** 6q24), all causing transient neonatal diabetes mellitus (TNDM), whereas mutations in mitochondrial DNA, such as the recurrent m.3243G > A, cause the maternally inherited diabetes and deafness (MIDD) [6, 7].

With the advent of novel DNA sequencing techniques (next generation sequencing; NGS), the simultaneous screening of coding sequences of all MDM genes has become possible. The screening can then be completed with additional methods that identify medium-large genetic deletions and methylation defects. As a result, many patients suspected of MDM can be genetically diagnosed and may benefit from tailored therapies [1, 6, 8, 9], expanding the aim and scope of pediatric diabetes clinics. Still, the amount of data produced by NGS may prove somehow overwhelming and a strict collaboration between geneticists and diabetologists with expertise in genetics may improve the pick-up rate of cases with robust genetic diagnosis. This in turn allows the appropriateness of customized therapies.

A Monogenic Diabetes Clinic (MDC) within the Diabetology and Growth Disorders Unit of Bambino Gesù Children’s Hospital was started by F.B. and N.R. on January 2019. The idea behind MDC was to convey patients suspected to have a monogenic form of diabetes or with an established genetic diagnosis of monogenic diabetes to a clinic exclusively designated for the diagnosis and care of this subtype of diabetes. The expected advantages of this organization are: 1) the implementation of a standardized pathway toward genetic testing, 2) to ease the revision of complex cases, 3) to administer standardized therapies for monogenic diabetes subtypes, 4) to gather rare cases of monogenic diabetes to the end of acquiring new knowledge on specific subtypes and 5) the identification of MDM in overweight or obese patients, easily diagnosed with type 2 diabetes mellitus (T2D) of youth [10, 11].

In this paper, we report the results of the first 3 years of activity of MDC.

## Materials and methods

### Patients

Patients with diabetes, impaired fasting glucose (IFG) or impaired glucose tolerance who tested negative for four types 1 diabetes (T1DM)-related autoantibodies (GADA, IA-2A, IAA, ZnT8) were considered eligible for genetic testing. Individuals with at least two independent fasting plasma glucose samples ≥ 100–125 mg/dl (5.6–6.9 mmol/L) were classified as IFG. Most, but not all IFG subjects underwent an oral glucose tolerance test (OGTT) to identify cases with diabetes or impaired glucose tolerance (IGT). A diagnosis of diabetes was established with two independent fasting plasma glucose samples ≥ 126 mg/dl (7.0 mmol/L) or one fasting plasma glucose sample > 126 mg/dl and a HbA1c value ≥ 6.5% (48 mmol/mol) or a value ≥ 200 at 120ʹ of OGTT or a random value ≥ 200 mg/dl. Age at onset of diabetes > 25 of the proband was not considered a criterium of exclusion for genetic testing if family history was indicative of autosomal dominant inheritance (3 consecutive generations). Presentation/accidental discovery of IFG/diabetes was in most cases between 0 and 18 years of age, with a few patients diagnosed beyond the age of 25. Parental history of IFG status or diabetes was obtained or actively established by requesting fasting plasma glucose analysis of parents. History of IFG/diabetes in both proband’s parents was not a criterium of exclusion. Lean patients with high fasting insulinemia (> 22 μU/ml) and acanthosis nigricans with or without fasting and/or post-load hyperglycemia were clinically classified as type A severe insulin resistance (SIR) and screened for insulin receptor (*INSR*) variants.

We were also consulted to give our opinion on previously identified monogenic diabetes genes defect/variant in two cases with no dysglycemia/diabetes. One patient was investigated for a mild intellectual impairment, while genetic test was requested for the other because of HbA1c repeatedly at the upper limit of reference range and a family history of type 2 diabetes.

Most patients were sent to MDC by physicians belonging to the Diabetology and Growth Disorders Unit of Bambino Gesù Children’s Hospital, but some patients were referred from hospitals outside Rome and Lazio region. A few patients self-referred to the clinic by word-of-mouth. Eight patients with non-autoimmune neonatal diabetes mellitus (NDM) from other centers were directly referred to F.B.

### T1DM autoantibodies

Autoantibodies were tested in the Clinical Laboratory Unit with ELISA commercial kits.

### Genetic screening

Clinical exome sequencing (CES, including 8245 genes) was performed on genomic DNA by using the Twist Custom Panel kit (Twist Bioscience, San Francisco, CA, USA) according to the manufacturer’s protocol on a NovaSeq6000 platform (Illumina, San Diego, CA, USA).

Coding sequences and intron/exon boundaries of the following genes were filtered out for analysis ("virtual panel"): *ABCC8, APPL1, CISD2, CNOT1, DNAJC3, DCAF17, KCNJ11, GCK, EIF2S2, EIF2AK3, INS, INSR, GATA4, GATA6, GLIS3, HNF1A, HNF4A, HNF1B, IER3IP1, NEUROD1, NEUROG3, GLIS3, PDX1, RFX6, MNX1, NKX2-2, PAX6, PCBD1, PTF1A, SLC2A2, SLC19A2, SLC29A3, EIF2S3, WFS1, ZFP57* [5].

The reads were aligned to human genome build GRCh37/UCSC hg19. Variant calling was performed with Dragen Germline Enrichment application of BaseSpace (Illumina, San Diego, CA, USA) while variant annotation and phenotype-based prioritization of candidate genes were carried out through the Geneyx Analysis software (Geneyx Genomex). A minimum depth coverage of 30X was considered suitable for analysis, **but** **most** **genes** **had** **a** **coverage** **of** **100X.** Exome sequencing data filtering was performed to identify protein-altering, putative rare recessive homozygous, compound heterozygous, and pathogenic or likely pathogenic heterozygous variants with an allele frequency < 1%. Variants were classified based on the guidelines of the American College of Medical Genetics and Genomics and the Association for Molecular Pathology (ACMG/AMP) [12]. Putative causative variants were analyzed by Sanger sequencing following a standard protocol (BigDye Terminator v3.1 Cycle Sequencing Kit, Life Technologies) to confirm the next-generation sequencing (NGS) results in probands, and, if possible, were investigated in the parents to check the inheritance status.

Patients with neonatal diabetes mellitus who resulted negative to the CES were analyzed for 6q24 aberrations by MS-MLPA analysis (ME033-A1, MrC Holland, Amsterdam, The Netherlands) that detects also methylation defects (6). Recently, two genes have been described as a novel causes of permanent neonatal diabetes: *ONECUT1* and *ZNF808* [13, 14]. These genes were examined in patients who were negative for standard screening in addition to *INS* promoter and *INS* intron 2 [5].

The report was fully explained and commented to the proband or to the proband’s guardians by F.B. and N.R. Reports regarding NDM cases were also discussed with caring physicians.

F.B. consulted with members of the Monogenic Diabetes Variant Curation Expert Panel (MDEP) [15] of Clinical Genome Resource [16] when novel variants in rare genes were identified.

## Results

**Fig. 1:**
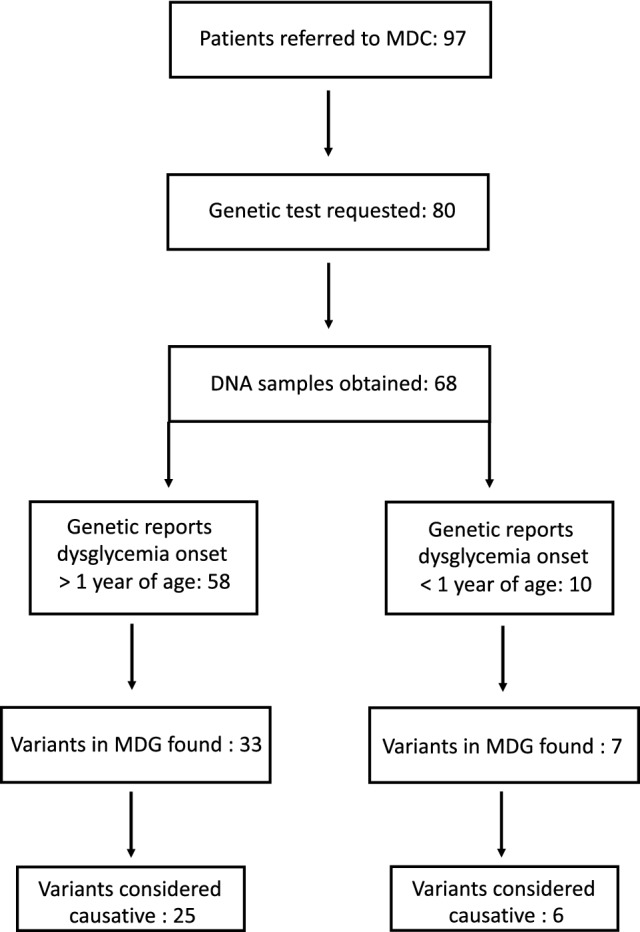
Steps from patient referral to MDC to final genetic diagnosis

A total of 97 patients were referred to the MDC during the 3-year (Jan/2019-Dec/2021) period. Four were from extra-European countries. Seventeen subjects did not fit inclusion criteria of MDM and no genetic test was requested. Blood sample for DNA extraction was not available for 12 patients at the time of writing. Therefore we were able to evaluate 68 genetic reports (Fig. 1).

Fiftysix incident cases were referred to investigate the origin of IFG, IGT or diabetes with onset beyond 1 year of age; in addition, we were requested to evaluate two normoglycemic cases carrying a MDG variant (Group 1, 58 cases) (Fig. 1). Among patients of this group, three lean females with fasting hyperinsulinism, hirsutism, acanthosis nigricans and (two cases) post-load glucose derangement were clinically classified as type A SIR. Only four patients of Group 1 were diagnosed with IFG, IGT or diabetes beyond 18 years of age. Ten patients (8 incident cases plus 2 past cases with permanent neonatal diabetes mellitus of unknown genetic cause) had diabetes onset within 1 year of age and were classified as neonatal diabetes mellitus (NDM; Group 2).

Among cases of Group 1, a pathogenic (P) or likely pathogenic (LP) variant of *GCK*, *HNF1A* and *HNF1B* was identified in 17, 4 and 1 patients, respectively (Table 1, Online resource 1, right panel). In a patient with liver adenomatosis carrying the *HNF1A*/Arg171Ter an additional somatic mutation has been also identified in the hepatic lesion.

**Table 1:** Variants that have been considered causative of the clinical phenotype: MODY, SIR, Fanconi-Bickel and NDM

Case	Gene	Age at diagnosis	Variant	ACGM (class)/ClinVar*	Hyperglycemia in parent/first degree relative	Variant search in relative	Proband status	Parent status
*GROUP 1*								
1	GCK	12 y, 4 m	c.775G > A; p.Ala259Thr	LP (IV)/ Conflicting interpretations	Father	Confirmed	DIABETES (OGTT)	DIABETES
2	GCK	8 y, 6 m	c.1373_1384delAGAAGGCCTGTA; p.Lys458_Cys461del	LP (IV)/n.r	Mother	Confirmed	IFG	IFG (p.c. GD)
3	GCK	12 y, 4 m	c.106C > T; p.Arg36Trp	P (V)/P	Father	Confirmed	IFG	IFG
4	GCK	5 y, 3 m	c.227C > T; p.Ser76Pro	LP (IV)/n.r	Mother	Confirmed	DIABETES	IFG (p.c. GD)
5	GCK	17 y, 5 m	c.501C > G; p.Trp167Ter	LP (IV)/P	Mother	Confirmed	IFG	IFG
6	GCK	17 y, 2 m	c.1312_1314delTTC; p.Phe438del	VUS (III)/n.r	Mother	Confirmed	IFG	IFG (p.c. GD)
7	GCK	15 y, 8 m	c.517G > T; p.Ala173Ser	LP (IV)/n.r	Father	Confirmed	IFG	IFG
8	GCK	3 y, 6 m	c.1331dupG; p.Ser445GlnfsTer14	LP (IV)/n.r	Mother	Confirmed	IFG	IFG (p.c. GD)
9	GCK	11 y	c.1396 T > G, p.Ter466GlyextTer144	VUS (III)/n.r	Father	Confirmed	DIABETES	DIABETES
10	GCK	10 y, 7 m	c.671 T > A; p.Met224Lys	LP (IV)/n.r	Mother	Confirmed	DIABETES	DIABETES (p.c. GD)
11	GCK	2 y, 4 m	c.821A > G; p.Asp274Gly	LP (IV)/n.r	Mother	non performed	IFG	p.c. GD
12	GCK	8 y	c.822C > A; p.Asp274Glu	LP (IV)/VUS	Mother	Confirmed	IFG	IFG (p.c. GD)
13	GCK	5 y, 8 m	c.501G > A; p.Trp167Ter	P (V)/P	Father	Confirmed	DIABETES	DIABETES
14	GCK	1 y, 11 m	c.667G > A; p.Gly223Ser	P (V)/P	Mother	Confirmed	IFG, IGT	IFG
15	GCK	6 y, 8 m	c.48_50delAGA; p.Glu17del	P (V)/VUS	Father	Confirmed	IFG	IFG
16	GCK	24 y, 10 m	c.480C > G; p.Ile160Met	LP (IV)/Conflicting interpretations	Daughter	Confirmed	IFG	IFG
17	GCK	10 y	c.1238A > T; p.Tyr413Phe	LP/n.r	Mother	Confirmed	IFG	IFG (p.c. GD)
18	HNF1A	14 y	c.735_736insGT; p.Ser247Cysfster96	LP (IV)/n.r	Mother	Confirmed	DIABETES (OGTT)	DIABETES
19	HNF1A	15 y	c.775G > A; p.Val259Ile	LP (IV)/VUS	Father	Confirmed	DIABETES	DIABETES
20	HNF1A	11 y	c.1146_1156del; p.Leu383AlafsTer32	LP (IV)/n.r	Mother	Confirmed	DIABETES (OGTT)	DIABETES
21	HNF1A	15 y, 7 m	c.511C > T; p. Arg171Ter	LP (IV)/P (expert panel)	Father	Confirmed	IGT	DIABETES
22	HNF1B	13 y	Exons 1–9 deletion	P (V)	NO	-	IFG, IGT	normoglycemia
23	INSR	15 y, 4 m	c.3492C > G; p.Asn1164Lys, spontaneous	P (V)/n.r	NO	Both negative	IGT	normoglycemia
24	INSR	12 y, 10 m	c.3473G > A; p.Arg1158Gln	LP (IV)/n.r	NOT REPORTED	Not performed	IGT	n.t
25	SLC2A2	16 y, 4 m	c.426G > A, p. Met142Ile/c.426G > A, p. Met142Ile	P (V)	Patient adopted	–	DIABETES	–
*GROUP 2 (incident cases)*								
26	KCNJ11	3 d	c.988 T > C; p.Tyr330His, spontaneous	P (V)	NO	Negative	PNDM	normoglycemia
27	KCNJ11	15 w	c.601C > T; p.Arg201Cys, spontaenous	P (V)	NO	Negative	PNDM	normoglycemia
28	KCNJ11	54 d	c.124 T > Co > A; p.Cys42Arg, paternal	P (V)	YES	Confirmed	TNDM	unknown
29	ABCC8	55 d	c.4136G > A; p.Arg1379His, paternal	P (V)	YES	Confirmed	TNDM	unknown
30	6q24	7 d	Methylation defect	n.a	NO	–	TNDM	normoglycemia
31	PDX1	1 d	c.[452C > T];[587A > C] (p.[Thr151Met], paternal;[Asn196Thr], maternal)	VUS (III)/ VUS (III)	NO	Confirmed	PNDM	normoglycemia

*MODY* Maturity onset diabetes of the young, *SIR* Severe insulin resistance, *NDM*  Neonatal diabetes mellitus, *P* Pathogenic, *LP*  Likely pathogenic, *VUS* Variant of uncertain significance, *IFG* impaired fasting glucose, *IGT*  Impaired glucose tolerance, *PNDM* Permanent neonatal diabetes mellitus, *TNDM* Transient neonatal diabetes mellitus, *n.a.*  not applicable, *n.t.*  not tested, *n.r.* not reported, *y*  year, *m* month, *d* day, *w*  week, *p.c.GD* previously classified as gestational diabetes. *ClinVar: https://www.ncbi.nlm.nih.gov/clinvar/

Four cases with a *GCK* variant (cases 4, 7, 8 and 17) and 1 with *HNF1A/*Arg171Ter were overweight. Three patients carrying the *GCK*/Ter466GlyextTer144 (case 9), the *HNF1A*/Ser247Cysfster96 variant (case 18) and the *HNF1B* exon 1–9 deletion (case 22) were obese. Interestingly, fasting C-peptide value of two latter patients was normal/high.

All patients with *GCK* or *HNF1A* heterozygous variants had a parent with IFG or diabetes who carried the same variant (Table 1). We considered causative two *GCK* variants of uncertain significance (VUS) (cases 6 and 9) because both proband and affected parent had a clinical phenotype (mild fasting, non-progressive hyperglycemia) consistent with *GCK* haploinsufficiency. Heterozygous pathogenic or likely pathogenic variants in the *INSR* were identified in 2 out of three patients with Type A SIR (Table 1, Online resource 1, right panel).

A homozygous variant of the *SLC2A2* gene (Fanconi-Bickel syndrome) was found in one patient (Case 25, Table 1). This case showed impaired glucose tolerance at the age of 2 years and diabetes at the age of 15 (oral glucose tolerance test: plasma glucose 262 mg/dl). Massive glycosuria was detected when the patient was 6 years old.

We did not formulate a final diagnosis in six cases belonging to Group 1 with a VUS variant (cases 33–36, 40) (Table 2 and Online resource 1, right panel). In addition, a *GCK* likely pathogenic variant was identified in a patient with no family history of diabetes who presented with diabetic ketoacidosis; in this case, the variant has not been considered sufficient to determine the clinical presentation (case 32; Table 2).

**Table 2:** Variants that have NOT been considered causative with available clinical data and cases bearing variants/genetic defects that might cause glucose derangement later on: MODY (case 38, 39), Type A severe insulin resistance (case 40)

Case	Gene	Age at diagnosis	Variant	ACGM (class)	Hypergly-cemia in parent	Variant search in parent	Clinical phenotype, ADA category	Parent status
Variants not causative of clinical phenotype								
32	GCK	4 y, 3 m	c.836A > G; p.Glu279Gly *	LP (IV)*	NO	-	T1D	normal
33	ABCC8	6 y, 10 m	c.2263C > T; p.Arg755Trp	VUS (III)	mother	confirmed	MODY, IFG	normal
34	PDX1	6 y, 11 m	c.755C > T, p.Ala252Val	VUS (III)	n.t	Not performed	MODY, Diabetes	n.t
35	KCNJ11	6 y, 6 m	c.820G > A; p.Asp274Asn	VUS (III) or LB (II)	father	Not performed	MODY, IFG	Diabetes
36	PDX1		c.97C > A; p.Pro33Thr	VUS (III)	father	Not performed	IFG	IFG
37	ABCC8	10 d	c.157A > T; p. Ser53Cys, maternal	VUS (III)	NO	confirmed	TNDM	normal
Variants that might cause glucose derangement later in life								
38	PDX1	13 y, 11 m	c.682_698delGCCGTGACCTCCGGCGA; p.Ala228GlyfsTer33	LP (IV)	father	Not performed (deceased)	normoglycemia	T2D (anecdotal)
39	HNF1B	9 y	arr 17q12(34,450,405–36,243,028) × 1 dn	n.a	NO	-	normoglycemia	-
40	INSR	12 y	c.2501G > A; p.Cys834Tyr	LP (IV)	unknown	Not performed	normoglycemia, fasting hyperinsulinemia in lean subject	mother: referred fasting hyper-insulinemia

*T1D* type 1 diabetes, *T2D*  type 2 diabetes, *MODY* Maturity onset diabetes of the young, *TNDM* Transient neonatal diabetes mellitus, *P* Pathogenic, *LP*  Likely pathogenic, *VUS*  Variant of uncertain significance, *LB* = likely benign, *IFG*  impaired fasting glucose, *n.t.*  not tested, *n.a.*  not applicable, *y*  year, *m*  month, *d* day

As part of MDC activity, we were consulted about two patients carrying a MDG variant, but with no defects of glucose metabolism at the time of writing (Table 2). The first one carries a heterozygous, frameshift *PDX1* likely pathogenic (LP) variant with premature termination codon (case 38), while the other has a large deletion of chromosome 17q12 encompassing *HNF1B* (case 39); of note, case 39 did not show any kidney malformation at ultrasound*.* For these two cases and for case 40 (*INSR* variant) we decided on a strict follow-up (every 6 months) in order to promptly diagnose any future derangement of glucose metabolism.

Twenty-five patients belonging to Group 1 were negative to genetic testing. Twelve had a parent with dysglycemia, (Table 3), while 13 were sporadic cases (Table 4). We formulated a diagnosis of T2D in two patients (cases 52 and 58) (Tables 3 and 4) who had normal, not declining c-peptide at onset of hyperglycemia and at follow-up. Other 2 (cases 64, 65) had a single diagnostic OGTT, leaving the T2D diagnosis dubious (Table 4). For cases 49, 51 (Table 3), 57, 60–63 (Table 4) our temptative diagnosis was autoantibody-negative T1D; however, we can not exclude an inherited (Table 3) or spontaneous (Table 4) small *HNF1A*/*HNF4A* deletion or pathogenic variant(s) in regulatory regions of these genes.

**Table 3:** Patients negative to genetic testing with a parent with glucose abnormalities

Case	Age at diagnosis	FPG (mg/dl); OGTT 120ʹ	C-peptide (ng/ml) at onset and at follow up	DKA	Current therapy	Type of diabetes, therapy of affected parent(s), generations with dyglycemia	Additional features. Temptative clinical diagnosis
**IFG,** **IGT**							
41	12 y, 8	116; 141	2.36	NO	None	Father T1D, Insulin; Mother GD; 2	IFG/IGT, at risk for T2D
42	11 y, 7 m	110; 134;	1.32	NO	None	Mother IFG, none; Father T2D, OHA; 2	IFG of unknown cause
43	19 y	102; n.a	n.a	NO	None	Father IFG, none; 2	IFG of unknown cause
44	10 y, 11 m	113; 164	n.a	NO	None	Mother GD; 3	IFG/IGT at risk for T2D
45	10 y, 9 m	106; 107	0.81	NO	None	Mother GD; 4	IFG of unknown cause
46	11 y, 1 m	112; 141	1.73	NO	None	Father T2D, n.a.; 3	IFG/IGT, at risk for T2D
47	11 y	103; 161	n.a	NO	OHA (Metformin)	Mother T2D, n.a.; 2	Overweight (BMI 23.4 whem 11 years old). IFG/IGT, at risk for T2D
48	13 y	77; 193	2.38	NO	None	Father T2D, OHA; 3	IGT, at risk for T2D
Diabetes, fasting							
49	25 y	343; n.a	n.a	NO	Insulin: 0.45/UI/kg/d	Mother GD; 2	Autoimmune tyroiditis. Autoantibody negative T1D
50	34 y	134; n.a	0.71	NO	n.a	Father; 3	Diabetes of unknown cause
51	6 y, 1 m	243; n.a	n.a	n.a	Insulin: 0.8/UI/kg/d	Father T1D, Insulin; 2	Autoantibody negative T1D
52	17 y	179; n.a	1.56; 1.35	NO	OHA (Metformin)	Mother T2D, OHA; 2	Lean (BMI 21.3 when 22 years old). T2D

*FPG*  fasting plasma glucose, *DKA* diabetic ketoacidosis, *y*  years, *m* months, *d* = day, *n.a.*  not available, *IU*  International Units, *GD* gestational diabetes, *T2D* type 2 diabetes, *T1D* type 1 diabetes, *IFG* impaired fasting glucose, OGTT, oral glucose tolerance test, *IGT* Impaired Glucose Tolerance, *BMI* Body Mass Index, *OHA* oral hypoglycemic agents

**Table 4:** Patients negative to genetic testing with negative parental history of glucose abnormalities

Case	Age at diagnosis, sex	FPG (mg/dl); OGTT 120ʹ	C-peptide (ng/ml) at onset and at follow up	DKA	Therapy	Additional features. Temptative clinical diagnosis
IFG or IFG/IGT						
53	17, M	117, 112	n.a	NO	none	IFG, unknown cause
54	13, F	120, 188	3.8	NO	none	IFG, IGT at risk for T2D
55	11, M	110, 143	n.a	NO	none	IFG, IGT at risk for T2D
56	12, F	116, 149	n.a	NO	none	IFG, IGT at risk for T2D
Diabetes, fasting						
57	13 y, M	307, n.a	0.32	NO	Insulin	T1D, autoantibody neg
58	10 y, F	532, n.a	3.05, 2.05	NO	Insulin	Overweight (BMI: 26.14 when 15 years old); T2D
59	13 y, M	192, n.a	1.03, 0.38	NO	Insulin	T1D, IA-2A positive after 1 year
60	15 y, M	n.a	n.a	DKA	Insulin	T1D, autoantibody neg
61	7 y, F	291, n.a	undetectable	NO	Insulin	T1D, autoantibody neg
62	12 y, F	362, n.a	0.55, 0.68	NO	Insulin	T1D, autoantibody neg
63	9 y, M	308, n.a	0.07	NO	Insulin	T1D, autoantibody neg
Diabetes, OGTT						
64	11 y, F	88, 232,	Basal Insulin: 135 μU/ml; C-pep: 4.92	NO	Metformin, 2 g/d	Overweight (BMI: 27.8 when 12 years old); Uric acid 6.1 mg/dl (2.4–5.7). Now normal weight. T2D ?
65	9 y, M	108, 226	2.01, 2.19	NO	Diet	Normal weight. T2D ?
Diabetes onset < 1 year of age						
66	54 w, M	715, n.a	0.39	NO	Insulin	Early onset, autoantibody neg. T1D
67	39 d, F	1033, n.a	undetectable	YES	Insulin	PNDM of unknown cause
68	34 w, M	530, n.a	n.a	YES	Insulin	Early onset, autoantibody neg. T1D

*FPG*  fasting plasma glucose, *DKA* diabetic ketoacidosis, *M* = male, *F* female, *y* years, *m* months, *w* weeks, *d*  days, *n.a.* not available, *T2D* type 2 diabetes, *T1D* type 1 diabetes, *IFG* impaired fasting glucose, *IGT* impaired glucose tolerance, *BMI* Body mass Index**,** *OGTT* oral glucose tolerance test

Six patients of Group 2 carried a pathogenic or likely pathogenic variant in *KCNJ11* (3 variants), *ABCC8* (1 variant) and *PDX1* (biallelic variants); a patient with transient neonatal diabetes mellitus had 6q24 methylation defects (Table 1; Online resource 1, left panel).

In a patient with TNDM the VUS *ABCC8/*Ser53Cys was identified (Table 2); the mother, carrying the variant, showed normal plasma glucose values at OGTT. We thus considered *ABCC8/*Ser53Cys not causative. This patient was also negative for *KCNJ11*, 6q24 and *SLC2A2*.

In three non-syndromic NDM cases (one incident, two past patients) we did not find any variant in the MDG of the panel or in the new NDM genes *ONECUT1* and *ZNF808.* Among these 3, two presented with diabetes beyond six months and before 1 year (cases 66, 68) and were classified as early-onset antibody-negative T1D (Table 4).

Therapeutic decisions consequent to genetic diagnosis are reported in Supplemental Table 1.

## Discussion

Next-generation sequencing has dramatically improved our capability of identifying even rare genetic causes of monogenic diabetes. Overall, genetic testing resulted positive and conclusive in 45.5% of cases (31/68). For patients of Group 1, the positive genetic testing rate was 43.1% (25/58 probands investigated) an acceptable percentage if compared to that obtained by the exceptionally meticulous services offered in the UK [3]. Among 36 patients of Group 1 with a parental history of hyperglycemia, 21 (Cases 1–21; Table 1) carried a heterozygous causative variant in *GCK* or *HNF1A,* with a pick-up rate of 58.3% (21/36) in this subgroup.

Among 22 cases with unknown or no parental history of hyperglycemia of Group 1, 3 carried a dominant variant and 1 a homozygous, recessive variant (Table 1, cases 22–25). All these cases have been investigated on the basis of specific clinical features: hyperinsulinism, hirsutism and acanthosis in lean females (cases 23 and 24), renal cysts (case 22), and tubular nephropathy (case 25). In one case with SIR, the *INSR* pathogenic variant arose spontaneously, while for the other proband parental DNA was not available for analysis. Therefore, among the subgroup lacking parental history, pick up rate was 18.1% (4/22). Recently, biallelic variants of *WFS1* have been identified in patients lacking syndromic features of Wolfram disease [17]. Moreover, biallelic *WFS1* pathogenic variants either syndromic or not, are quite frequent in pediatric patients born to consanguineous parents, where autosomal recessive mutations represent more than 40% [18]. However, no WFS1 variant was identified in this series. In contrast, a *SLC2A2* homozygous variant was found in a single patient, setting the frequency of recessive mutations of Group 1 to 1.7% (1/58) or 4.5% (1/22) when considering the subgroup of patients with mute family history of diabetes. Though based on a relatively small number of patients, it seems reasonable to conclude that genetic testing in individuals with onset of hyperglycemia beyond 1 year of age and without parental history of hyperglycemia should be mainly reserved for cases with extrapancreatic features and/or consanguineous parents [19]. Interestingly, but not surprisingly, autosomal dominant -negative mutations of *INSR* are not found in populations with high consanguinity rate [18] but can be identified in probands lacking parental history of hyperglycemia (our two cases) on the basis of extrapancreatic features [20].

Follow-up was recommended for cases 38, 39 and 40 who are currently normoglycemic (Table 2). Case 38 bears a 17q12 deletion that includes HNF1B; this abnormality arises spontaneously in 70% of cases and has been associated with high frequency of diabetes (63%) with onset in adulthood [21]. Our case showed abnormally high glucose levels at 30ʹ of OGTT, indicating a poor first-phase insulin secretion, a finding that is in keeping with those of Ng et al. that indicate a marked insulin deficiency in *HNF1B* patients [22]. Case 39 underwent genetic testing because of strong family history of diabetes from the paternal side (father deceased). She carries a likely pathogenic *PDX1* variant (Ala228GlyfsTer33) which is similar to *PDX1*/Pro63ArgfsTer60, the only *PDX1* variant linked to diabetes with well demonstrated dominant-negative effect [23]. *INSR* VUS detected in case 40 may concur with fasting hyperinsulinemia but without functional data, it is not possible to opt for a dominant-negative effect.

It is well established that NDM is quite rare (about 1:100,000 live births) in populations with low consanguinity rate [24]. Nine NDM patients out of 10 in the present study were not syndromic and in 6 a causative variant was identified, including a patient with pancreas hypoplasia linked to biallelic PDX1 variants (case 31). Two new NDM genes [13, 14] were additionally screened in the remaining four with no success. Of interest, we found a heterozygous, LP missense variant of *ONECUT1* in another individual diagnosed with PNDM 18 years ago and not included in the present study [25].

A genetic diagnosis may guide therapeutic changes (Supplemental Table 1) such as switch from insulin to sulfonylureas (SU) in patients with NDM due to *KCNJ11* or *ABCC8* (ATP-sensitive potassium channel genes, K_ATP_) variants [8, 9]. We attempted the transfer to glibenclamide (the most used SU in neonatal diabetes) all NDM patients with K_ATP_ variants and succeeded in 3, while one patient was resistant even at high glibenclamide dosage [26]. Metformin was introduced after genetic diagnosis in one patient with INSR variant (case 24). Individuals with type A insulin resistance may show severe hyperglycemia over time [27]; however, we do not know whether the early use of metformin in case 23 may prevent the onset of full-blown diabetes later in life. Recently, new therapies aimed at handling hyperinsulinemia and diabetes seen in patients with congenital SIR due to INSR mutations and in type A SIR have been proposed [28, 29].

An attempt to transfer to SU *HNF1A* patients treated with insulin is common practice [1]. However, the patient with liver transplant (case 21) recently stopped insulin, while case 19 continued insulin because she became pregnant. Metformin was confirmed by the caring physician in an obese patient after *HNF1A* diagnosis (case 18), while the obese (BMI 30.8) patient carryng the *HNF1B* deletion (case 22) is on diet. Obesity "complicating" MODY [10, 11] is becoming a frequent issue in Italian patients. Intriguingly, also the affected parent of *HNF1A* case was obese, making clinical diagnosis even more complex.

Among limitations of this work, there is the very small size of our cohort. Nevertheless, results are in line with those obtained by others and positive diagnostic rate was acceptable in cases with parental history of hyperglycemia. The second limitation is that we did not analyze genes causing lipodystrophies and the frequent mitochondrial mutation causing maternally inherited diabetes and deafness (MIDD), *m.3243A* > *G*. As for *m.3243A* > *G*, while this mutation is a relatively common cause of hyperglycemia in data sets of adult-onset diabetes [3, 7], it seems to be very rare in the pediatric setting [18].

## Conclusions

In conclusion, during the first 3 years of activity MDC seemed to fulfill the objectives that were set at its start, especially points 2, 3 and 5 described in the introduction. A slight change in strategy for selection of "sporadic" cases with non-autoimmune diabetes, focused on thorough, systematic workup of expancreatic features, will be implemented in future MDC activities.

## Supplementary Information

Below is the link to the electronic supplementary material.

**Supplementary file1** Center pie chart shows incident NDM cases (in orange), 57 cases with clinical diagnosis of MODY plus  2 past cases with NDM (in blue) and cases with clinical diagnosis of type A severe insulin resistance (in grey). Pie chart on the left shows pathogenic, likely pathogenic and variants of uncertain significance (VUS) identified in incident patients with NDM. Pie chart on the right shows MODY cases positive to GCK (in orange), HNF1A (in grey), INSR (in dark blue) or causative variants in other genes (in red). In light blue variants of undetermined effect and in 2 shades of green cases negative to genetic testing. (PDF 37 KB)

Supplementary file2 (DOCX 22 KB)

## Data Availability

Data obtained with the genetic analysis are not archived publicly.
