# Correction to: Monogenic diabetes clinic (MDC): 3‑year experience

**DOI:** 10.1007/s00592-022-01998-6

**Published:** 2022-11-05

**Authors:** Novella Rapini, Patrizia I. Patera, Riccardo Schiaffini, Paolo Ciampalini, Valentina Pampanini, Matteoli M. Cristina, Annalisa Deodati, Giorgia Bracaglia, Ottavia Porzio, Rosario Ruta, Antonio Novelli, Mafalda Mucciolo, Stefano Cianfarani, Fabrizio Barbetti

**Affiliations:** 1 Diabetology and Growth Disorders Unit, Bambino Gesù Children’s Hospital, IRCCS, 00164 Rome, Italy; 2 Clinical Laboratory Unit, Bambino Gesù Children’s Hospital, Piazza S Onofrio 4, 00165 Rome, Italy; 3 Department of Experimental Medicine, Univerisity of Rome ‘Tor Vergata’, 00131 Rome, Italy; 4 Translational Cytogenomics Research Unit, Bambino Gesù Children’s Hospital, IRCCS, 00146 Rome, Italy; 5 Department of Systems Medicine, University of Rome ‘Tor Vergata’, 00131 Rome, Italy; 6 Department of Women’s and Children’s Health, Karolinska Institutet, 17177 Stockholm, Sweden

## Correction to: Acta Diabetologica 10.1007/s00592-022-01972-2

Author would like to update below listed corrections.

1. In page 2 under “**Genetic screening”** all gene names to be deleted that were reported twice, namely: CISD2, PDX1, EIF2AK3, PTF1A, IER3IP1, CNOT1 and SLC19A2.
2. Table 2 caption, numbers in parenthesis to be changed as: 37 should read 38, 38 should read 39 and 39 should read 40.

The original article has been corrected.

