# Supplementary material for: Monogenic diabetes clinic (MDC): 3-year experience
Source: Acta Diabetol. 2022 Sep 30;60(1):61–70. doi: 10.1007/s00592-022-01972-2 (PMC9813184; doi:10.1007/s00592-022-01972-2)
Supplement: Supplementary file 1 — Supplementary file1 Center pie chart shows incident NDM cases (in orange), 57 cases with clinical diagnosis of MODY plus 2 past cases with NDM (in blue) and cases with clinical diagnosis of type A severe insulin resistance (in grey). Pie chart on the left shows pathogenic, likely pathogenic and variants of uncertain significance (VUS) identified in incident patients with NDM. Pie chart on the right shows MODY cases positive to GCK (in orange), HNF1A (in grey), INSR (in dark blue) or causative variants in other genes (in red). In light blue variants of undetermined effect and in 2 shades of green cases negative to genetic testing. (PDF 37 KB) [file 592_2022_1972_MOESM1_ESM.pdf]

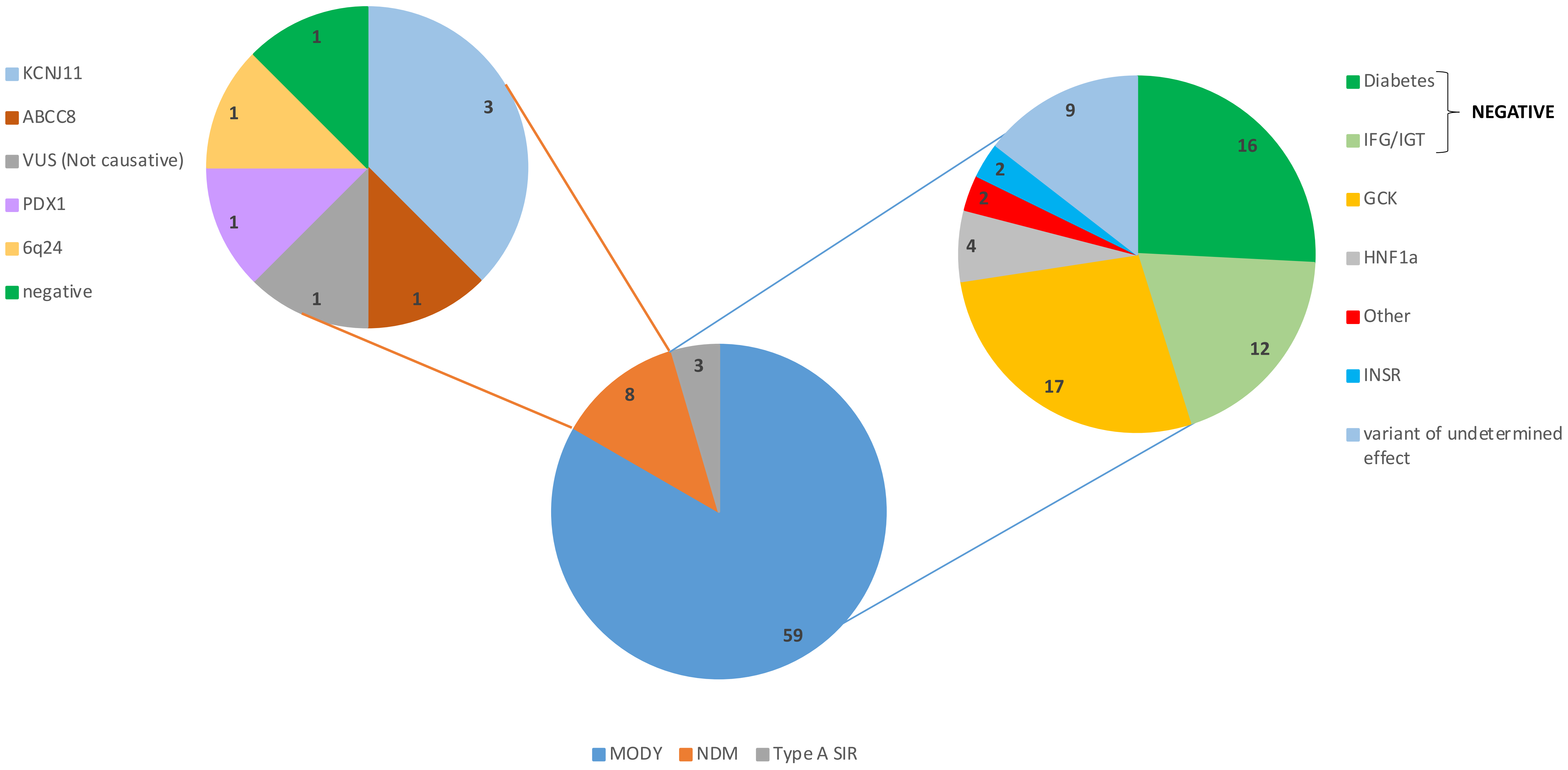

KCNJ11
ABCC8
VUS (Not causative)
PDX1
6q24
negative
1
3
1
1
1
1
Diabetes
IFG/IGT
NEGATIVE
GCK
HNF1a
Other
INSR
variant of undetermined effect
9
16
2
2
4
17
12
3
8
59
MODY
NDM
Type A SIR
