# Supplementary material for: Monogenic diabetes clinic (MDC): 3-year experience
Source: Acta Diabetol. 2022 Sep 30;60(1):61–70. doi: 10.1007/s00592-022-01972-2 (PMC9813184; doi:10.1007/s00592-022-01972-2)
Supplement: Supplementary file 2 — Supplementary file2 (DOCX 22 KB) [file 592_2022_1972_MOESM2_ESM.docx]

| **Supplemental Table 1. Therapy before and after genetic testing** | | | | | | | | |
| --- | --- | --- | --- | --- | --- | --- | --- | --- |
| **Case** | **Gene** | **Age at diagnosis** | **Variant** | **CLINICAL DIAGNOSIS** | **Therapy: before** | **HbA1c before (mmol/mol)** | **Therapy: after** | **HbA1c after (mmol/mol)** |
| 1 | GCK | 12 y, 4 m | p.Ala259Thr | MODY | none | 39 | none | 41 |
| 2 | GCK | 8 y, 6 m | p.Lys458_Cys461del | MODY | none | 43 | none | 42 |
| 3 | GCK | 12 y, 4 m | p.Arg36Trp | MODY | none | 46 | none | 45 |
| 4 | GCK | 5 y, 3 m | p.Ser76Pro | MODY | none | 41 | none | 42 |
| 5 | GCK | 17 y, 5 m | p.Trp167Ter | MODY | none | 46 | none | 50 |
| 6 | GCK | 17 y, 2 m | p.Phe438del | MODY | none | 45 | none | 44 |
| 7 | GCK | 15 y, 8m | p.Ala173Ser | MODY | none | 50 | none | n.a. |
| 8 | GCK | 3 y, 6 m | p.Ser445GlnfsTer14 | MODY | none | 44 | none | 50 |
| 9 | GCK | 11 y | p.Ter466GlyextTer144 | MODY | none | 49 | none | 51 |
| 10 | GCK | 10 y, 7 m | p.Met224Lys | MODY | none | 40 | none | 42 |
| 11 | GCK | 2 y, 4 m | p.Asp274Gly | MODY | none | 43 | none | 46 |
| 12 | GCK | 8 y | p.Asp274Glu | MODY | none | 48 | none | 47 |
| 13 | GCK | 5 y, 8 m | p.Trp167Ter | MODY | none | 45 | none | 46 |
| 14 | GCK | 1 y, 11 m | p.Gly223Ser | MODY | none | 46 | none | 47 |
| 15 | GCK | 6 y, 8 m | p.Glu17del | MODY | insulin | 42 | insulin (0.06 U/kg/d) | 40 |
| 16 | GCK | 21 days | p.Ile160Met | MODY | none | n.t. | none | n.a. |
| 17 | GCK | 10 y | p.Tyr413Phe | MODY | none | 36 | none | 38 |
| 18 | HNF1A | 14 y | p.Ser247Cysfster96 | MODY, obese (BMI:30 when 20 y old) | Metformin 850 mg t.i.d. | 52 | Metformin 850 mg t.i.d. | 51 |
| 19 | HNF1A | 15 y | p.Val259Ile | MODY | Insulin | 61 | Insulin | 41 |
| 20 | HNF1A | 11 y | p.Leu383AlafsTer32 | MODY | None | 40 | None | 41 |
| 21 | HNF1A | 15 y, 7 m | p. Arg171Ter | MODY, liver adenomatosis, liver transplant, overweight (BMI: 26.1 when 22 y old) | Metformin | 40 | Metformin | 36 |
| 22 | HNF1B | 13 y | Exons 1-9 deletion | MODY, renal cysts | Diet | 35 | Diet | 36 |
| 23 | INSR | 15 y, 4 m | p.Asn1164Lys | SIR | Metformin 500 mg b.i.d. | 34 | Metformin 500 mg b.i.d. | 35 |
| 24 | INSR | 12 y, 10 m | p.Arg1158Gln | SIR | none | 28 | Metformin 250 mg b.i.d. | 29 |
| 25 | SLC2A2 | 16 y, 4 m | p.Met142Ile/p.Met142Ile | FBS | none | /// | deceased | /// |
| 26 | KCNJ11 | 3 d | p.Tyr330His, spontaneous | PNDM | Insulin | n.t. | Insulin (SU resistant) | 64 |
| 27 | KCNJ11 | 15 w | p.Arg201Cys, spontaenous | PNDM | Insulin | n.t. | Glibenclamide | 46 |
| 28 | KCNJ11 | 54 d | p.Cys42Arg, paternal | TNDM | Insulin | n.t. | Glibenclamide | n.t. |
| 29 | ABCC8 | 55 d | p.Arg1379His, paternal | TNDM | Insulin | n.t. | Glibenclamide | n.t. |
| 30 | 6q24 | 7 d | Methylation defect | TNDM | Insulin | n.t. | none | n.t. |
| 31 | PDX1 | 1 d | p.Thr151Met/Asn196Thr | PNDM | Insulin | n.t. | Insulin | n.t. |
| 32 | GCK | 4 y, 3 m | p.Glu279Gly | T1D; 3rd grade AV block, pace maker. | Insulin | HbA1c unknown, FPG at onset: 384 mg/dl, pH 7.18 | lost at follow up | /// |

Table 3 legend: MODY= Maturity Onset Diabetes of the Young; SIR= Severe Insulin Resistance; PNDM= permanent neonatal diabetes mellitus; TNDM= transient neonatal diabetes mellitus; FBS=Fanconi-Bickel syndrome; n.t.= not tested; y=year; m=month; d=day; w=week; FPG=fasting plasma glucose.
